# Potential Therapeutic Benefits of Herbs and Supplements in Patients with NAFLD

**DOI:** 10.3390/diseases6030080

**Published:** 2018-09-10

**Authors:** Brandon J. Perumpail, Andrew A. Li, Umair Iqbal, Sandy Sallam, Neha D. Shah, Waiyee Kwong, George Cholankeril, Donghee Kim, Aijaz Ahmed

**Affiliations:** 1 Drexel University College of Medicine, Philadelphia, PA 19129, USA; brandonperumpail@gmail.com; 2 Department of Medicine, Stanford University School of Medicine, Stanford, CA 94305, USA; andrewli@stanford.edu; 3 Department of Medicine, Mary Imogene Bassett Hospital, Cooperstown, NY 13326, USA; umairiqbal_dmc@hotmail.com; 4 Division of Gastroenterology and Hepatology, Stanford University School of Medicine, Stanford, CA 94305, USA; SSallam@stanfordhealthcare.org (S.S.); NeShah@stanfordhealthcare.org (N.D.S.); WKwong@stanfordhealthcare.org (W.K.); georgetc@stanford.edu (G.C.); dhkimmd@stanford.edu (D.K.)

**Keywords:** NAFLD, NASH, nonalcoholic fatty liver disease, herb, silymarin, resveratrol, green tea, coffee, turmeric, garlic, *Ginkgo biloba*, cannabinoids, fish oil

## Abstract

Our aim is to review the efficacy of various herbs and supplements as a possible therapeutic option in the treatment and/or prevention of nonalcoholic fatty liver disease (NAFLD). We performed a systematic review of medical literature using the PubMed Database by searching the chemical names of many common herbs and supplements with “AND (NAFLD or NASH)”. Studies and medical literature that discussed the roles and usage of herbs and supplements in NAFLD and nonalcoholic steatohepatitis (NASH) from inception until 20 June 2018 were reviewed. Many studies have claimed that the use of various herbs and supplements may improve disease endpoints and outcomes related to NAFLD and/or NASH. Improvement in liver function tests were noted. Amelioration or reduction of lobular inflammation, hepatic steatosis, and fibrosis were also noted. However, well-designed studies demonstrating improved clinical outcomes are lacking. Furthermore, experts remain concerned about the lack of regulation of herbs/supplements and the need for further research on potential adverse effects and herb–drug interactions. In conclusion, preliminary data on several herbs have demonstrated promising antioxidant, anti-inflammatory, anti-apoptotic, and anti-adipogenic properties that may help curtail the progression of NAFLD/NASH. Clinical trials testing the safety and efficacy must be completed before widespread use can be recommended.

## 1. Introduction

Nonalcoholic fatty liver disease (NAFLD) and its progressive subset, nonalcoholic steatohepatitis (NASH), affect a significant proportion of the population, with no approved drug treatment. The current treatment option is lifestyle modification through diet and exercise [1,2,3]. In this special issue, the discussion on potential drug targets, oral hypoglycemics, lipid-lowering agents, vitamins/minerals, and surgical options will be reviewed in other sections. The focus of this article is to review the role of herbs and supplements in patients with NASH, the less common but aggressive subset of NAFLD. 

Alternative medicine has recently gained interest as a possible approach to disease therapy [4,5]. Herb usage is popular in eastern medicine, especially in countries such as India and China, and it is becoming increasingly popular in the Western hemisphere [4,5]. Many people are looking to herbs and supplements as a natural alternative to drugs. Herbs have many great antioxidant, anti-inflammatory, anti-apoptotic, and anti-adipogenic effects (decreased adipose tissue formation and weight loss) that allow them to be possible therapeutic agents in NAFLD treatment [4,5].

## 2. Herbs Studied with NAFLD

### 2.1. Milk Thistle

Silymarin is the extract from the milk thistle plant (*Silybum marianum*); both *Silybum* and silymarin have been consistently studied with respect to NAFLD and have been shown to have therapeutic effects [6,7,8,9,10,11,12,13,14,15,16,17,18,19,20,21,22,23,24]. Silymarin has been used for centuries as a natural remedy for liver disease [10]. Silymarin is a powerful antioxidant in the cell that can increase catalase and glutathione levels and also scavenge free lipid peroxyl radicals in hepatocytes [6,10,12,17,19,22,25]. Through its antioxidative capabilities, silymarin can restore NAD^+^ homeostasis, Sirtuin 1 (SIRT1) activity, and the AMP-activated protein kinase α (AMPKα) pathway to improve poly-(ADP-ribose)-polymerase (PARP) function, which protects the cell from the oxidative damage observed in NAFLD [22]. This effect also allows for improved hepatic lipid homeostasis [17,22]. This herb can also reduce hepatic de novo lipogenesis through the downregulation of peroxisome proliferator-activated receptor γ (PPARγ), acetyl-CoA carboxylase (ACC), and fatty acid synthase (FAS) [18]. Furthermore, silymarin can reduce steatosis and insulin resistance seen in NAFLD through restoration of the insulin receptor substrate-1 (IRS-1)/PI3K/Akt pathway [12]. Silymarin is also able to reduce hepatic inflammation through activation of the farnesyl X receptor (FXR) which correlates with the inhibition of NF-κB transactivity [12,16]. Due to the vast data supporting milk thistle as a therapeutic option from experimental mouse studies, there are multiple clinical trials testing its effects [7,9,11,12,16,17,18,20,21,22]. In a randomized double-blinded, placebo-controlled trial, Wah Kheong et al. tested the effects of silymarin supplementation on adult patients with biopsy-proven NASH and a NAFLD activity score (NAS) of greater than 4 [23]. The 99 patients were divided into a treatment group that received 700 mg three times daily and a placebo group [23] for 48 weeks [23]. The treatment group showed reduced aspartate aminotransferase with reduced fibrosis and liver stiffness without any issues in tolerability in patients [23]. Another study by Solhi et al. treated NASH patients with 210 mg of silymarin daily and after 8 weeks noted decreased alanine aminotransferase (ALT) and aspartate amino transferase (AST) levels in the treated group [13]. Zhong et al. completed a meta-analysis of eight randomized controlled trials (RCTs) studying NAFLD, and concluded that silymarin had statistically significant effect in reducing AST and ALT levels in patients [24].

### 2.2. Resveratrol

Resveratrol is another common alternative treatment studied with NAFLD [26,27,28,29,30,31,32,33,34,35,36]. Resveratrol has been shown to have similar effects to milk thistle in that is has antioxidative and anti-inflammatory properties [26,27,28,29,30,31,32,33,34,35,36]. Resveratrol supplementation is able to decrease inflammation and decrease hepatic steatosis through the inhibition of overactivated inflammatory agent NF-κB through the activation of the AMPKα/SIRT1 pathway which also regulates hepatic lipid metabolism and allows for lipid accumulation clearing through autophagy [31]. Resveratrol has also been shown to help decrease endoplasmic reticulum (ER) stress markers such as C/EBP homologous protein (CHOP) [33]. ER stress has been linked with NAFLD, lipotoxicity, hepatocyte apoptosis, and steatosis [33]. Sterol regulatory element-binding proteins (SREBPs) are key for de novo lipogenesis, and over-activation of this process is exhibited in NAFLD [36]. Resveratrol can block the activation of SREBP-1 and SREBP-2 by inhibiting their proteolytic cleavage [36]. Also, by inhibiting the carnitine palmitoyltransferase (CPT-1) and uncoupling protein (UCP-2), resveratrol can decrease free fatty acid β-oxidation and the production of reactive oxygen species (ROS) [36]. Although the experimental data on resveratrol supports its use with NAFLD, an early study found that there was no benefit to resveratrol supplementation [26,31,33,35,36]. This early study by Chachay et al. gave 10 obese NAFLD patients 3000 mg of resveratrol for 8 weeks, noting no improvement and even increased liver enzymes [26]. However, more recent studies have indicated that resveratrol could be a therapeutic agent for NAFLD patients [27,28,29,30,32,34]. In a double-blinded RCT by Faghihzadeh et al., 50 NAFLD patients were divided to receive lifestyle modification and placebo or 500 mg of resveratrol for 12 weeks and after the study period the treatment group had improved inflammatory cytokines, ALT, and hepatic steatosis compared to the placebo group [27,29]. A similar RCT by Chen et al. with 60 NAFLD patients randomized to either placebo or 600 mg of resveratrol for three months noted improved levels of ALT, AST, insulin resistance, and inflammatory factors [28]. Furthermore, Heeboll et al. observed that dosing affected the efficacy of resveratrol as a treatment [30]. Their study noted that high-dose resveratrol does not improve NAFLD histologically but has minimal ameliorating effects on liver enzymes [30]. Despite the beneficial effects of resveratrol in the clinical studies mentioned, meta-analysis by Elgebaly et al. concluded that current research is insufficient to claim that resveratrol improves NAFLD fibrosis and did not significantly improve liver enzymes [34].

## 3. Caffeine and Tea

### 3.1. Coffee

Consumed daily by many individuals, coffee has been noted as having an inverse relationship with NAFLD disease progression in many studies [37,38,39,40,41,42,43,44,45,46,47,48]. A cross-sectional study by Bambha et al. analyzed coffee drinking habits in 782 patients in the NASH Clinical Research Network and correlated coffee intake with decreased odds of advanced fibrosis in patients with lower insulin resistance [38]. In a similar study, Zelber-Sagi et al. examined cross-sectional as well as prospective patient data for 7 years in a sub cohort of patients [41]. In the cross-sectional portion of the study, increased coffee intake was inversely associated with clinically significant fibrosis [41]. Furthermore, the prospective analysis noted no association between new development of NAFLD and coffee intake but observed a protective effect of coffee on fibrosis progression [41]. When comparing patients with NAFLD to healthy individuals, Gutierrez-Grobe et al. noted that coffee intake was significantly higher in the healthy patients compared to those with NAFLD [49]. Coffee can upregulate ER and mitochondrial chaperone proteins such as glucose-related protein 78 (GRP78) which modulates ER homeostasis and prevents the activation of SREBP-1 [39]. Experimental studies with GRP78 knockout mice noted increased inflammation, steatosis, and apoptosis in hepatocytes due to its role in the unfolded protein response [50]. Coffee, contains polyphenols similar in structure to silymarin, which can also increase the production of antioxidant proteins [39]. Coffee notably increases periredoxin-1 (PRDX-1) which helps reduce ROS and decrease the oxidative stress in hepatocytes [39]. The synergistic effect of the polyphenols and caffeine in hepatocytes allow it to decrease insulin resistance and steatohepatitis [46]. Moreover, the polyphenols seem to be the cause of the antifibrotic effects of coffee over caffeine [46]. Finally, meta-analysis conducted by Wijarnpreecha et al. noted that coffee drinkers had a decreased risk of NAFLD and that regular daily consumption of coffee correlated with decreased risk of fibrosis [47].

### 3.2. Green Tea

Green tea, made from the leaves of the *Camillia sinensis* plant, like coffee, is commonly consumed and has been well documented to have beneficial effects with NAFLD [51,52,53,54,55,56,57,58,59,60,61,62]. The main therapeutic agent for NAFLD in green tea is epigallocatechin-3-gallate (EGCG) [54,55]. EGCG decreases hepatic inflammation through the reduction of hepatic cyclooxygenase-2 (COX2), prostaglandin E2, NF-κB, and toll-like receptor 4 (TLR4) [53,56,59]. EGCG also regulates hepatic lipid homeostasis through modulation of mitochondrial complex chain proteins and the previously mentioned AMPK, IRS-1, SREBP, and PPARγ pathways [54,55,60,61]. Furthermore, the polyphenols and EGCG in green tea allow for an antioxidative effect through NADPH oxidase and cytochrome P450 2E1 (CYP2E1) [62,63]. In the clinical setting, many trials have seen improvements in NAFLD patients with green tea therapy [51,52,57,58]. A double-blinded RCT with 80 NAFLD patients by Pezeshki et al. noted improved AST and ALT in patients supplemented with 500 mg of green tea extract daily compared to placebo [57]. Another study 12-week study by Hussein et al. with 80 patients randomized to receive 500 mg of green tea extract or placebo daily noted improvement in all parameters of NAFLD measured in the treatment group [58]. The green tea group observed improved inflammatory markers, aminotransferases, insulin resistance, adiponectin, and regression of fatty liver on ultrasound examination [58].

## 4. Household Herbs

### 4.1. Turmeric

Of all the common household spices and herbs, turmeric and garlic have the most research in the setting of treatment. Turmeric (active ingredient curcumin), also known as the Siam tulip, is a plant most notably cultivated in India as a spice recognized by its yellow color. Turmeric has been studied to be a therapeutic agent for NAFLD patients [64,65,66,67,68,69,70,71,72,73,74,75,76,77,78]. Curcumin is acknowledged as powerful antioxidant and anti-inflammatory agent, in addition to its many other beneficial features [74]. It acts as an antioxidant through the ability to neutralize free radicals and ROS that lead to oxidative stress in cells via the nuclear factor E2-related factor (Nrf2) and NADPH oxidase [68,74,77,78]. Curcumin can decrease major NAFLD risk factors such as hyperuricemia, dyslipidemia, and insulin resistance through the reduction of serum lipids and uric acid concentration [66]. It can also decrease hepatic steatosis through lipid homeostasis by increasing the inhibition of CD36 and PPARγ by the cAMP response element-binding (CREB) protein and the adiponectin precursor, ADIPOQ [64,72,77]. Curcumin can enhance the expression of PPARα and liver X receptor alpha (LXRα) and reduce SREBP activation and improve lipid balance [76,77]. Hepatic inflammation is controlled by the ability of curcumin to regulate the levels of hepatic proteins NF-κB, IP-10, IL-1β, IFN-γ, TLR4, and CD68 [68]. Also, the impairment intestinal mucosal mechanical barrier typical of NASH is reduced through curcumin’s upregulation of occludin [70]. Clinical trials have been used to confirm the experimental benefits of curcumin use in NAFLD patients [66,67,71,73,75]. Panahi et al. studied 102 NAFLD patients randomized to receive placebo or 1000 mg/day of curcumin for 8 weeks and concluded that curcumin improved hepatic steatosis determined through imaging and liver enzymes in NAFLD patients without any issues of tolerance [73]. A prospective cohort study by Selmanovic et al. with 400 mg curcumin supplementation also noted statistically significantly improved liver ultrasound results [74].

### 4.2. Garlic

Garlic is one of the most common herbs used around the world in cooking and medicine [79,80,81,82,83,84,85,86]. The main active components that give rise to the therapeutic effects of garlic are S-allyl cysteine (SAC), S-allymercaptocysteine (SAMC), diallyl disulfide (DADS), and cinnamoyloctopamines [79,80,81,82,83,84,85,86]. SAC is very similar to resveratrol is its ability to act as a SIRT1 activator that induces the activation of the AMPKα pathway and reduces hepatic lipogenesis and lipotoxicity [79]. It can also prevent lipid induced cell death by reducing free fatty acid ROS production and caspase activation [79]. SAMC and DADS act as antioxidants by inhibiting CYP2E1 and increasing catalase and glutathione peroxidase (GPx) [80,82]. Furthermore, it can decrease fibrosis factors, Transforming Growth Factor-β1 (TGF-β1) and α-Smooth Muscle Actin (α-SMA), and the inflammatory cytokines that would lead to the activation hepatic Kupffer cells and hepatic stellate cells (HSCs) that would cause collagen deposition [80]. Finally, SAMC can reduce inflammation through NF-κB and improve lipid homeostasis and insulin resistance through the AMPK and IRS-1/PI3K/Akt pathway [80,81]. DADS is also able to provide therapeutic effects through the stimulation of PPARα and CPT-1 [82]. The cinnamoyloctopamines are antioxidants that increase superoxide dismutase (SOD) and anti-inflammatory factors by decreasing COX-2 [84]. Clinically, Soleimani et al. noted that 800-mg garlic supplementation in NAFLD patients was able to significantly decrease body weight and body fat mass [85]. Furthermore, a double blinded RCT by Kim et al. observed improved ALT in patients with mild hepatic dysfunction who were given fermented garlic extract [86]

### 4.3. Rosemary, Peppermint, Basil, Lavender, Oregano, and Sage 

Ursolic acid and carnosic acid are the active components of many herbs used in the common household [87,88]. Ursolic acid is found in rosemary, peppermint, basil, lavender, and oregano while carnosic acid is in rosemary and sage [87,88]. Ursolic acid’s ability to improve lipotoxicity and lipid homeostasis comes through regulation of PPARα, the AMPK pathway, and reduction of ER stress in hepatic cells [87,89,90]. Carnosic acid acts through modulation of hepatic SOD, SIRT1, NF-κB, PI3K/Akt, and SREBP-1c to exert antioxidant, anti-inflammatory, anti-adipogenic, and anti-apoptotic effects [88,91,92,93,94]. Further research on these herbs is necessary to determine their effects on NAFLD patients.

### 4.4. Ginger, Cocoa, and Cinnamon

Ginger, cocoa, and cinnamon have been explored as potential therapeutic agents, but not much is known about their use yet. Of the three, most of the research supports ginger supplementation [95,96,97]. Ginger has antioxidative capabilities through the inhibition of CYP2E1 and upregulation of SOD, catalase, and GPx to reduce ROS and oxidative stress [96] Furthermore, ginger has anti-inflammatory capabilities by reducing cytokines Tumor Necrosis Factor-α (TNF-α), IL-1β, and IL-6, as well as anti-lipotoxic/lipogenic capabilities by reducing SREBP-1c, ACC, and FAS [96]. A NAFLD 44-patient study by Rahimlou et al. observed improved ALT, insulin resistance, and hepatic steatosis in patients receiving two grams of ginger supplementation daily for 12 weeks versus placebo [97]. One particular type of ginger to be considered is *Alpinia zerumbet* [98,99]; which is a tropical plant commonly used as a Japanese herbal medicine and has been associated with the longevity of the people of Okinawa, Japan [98]. Its main component is dihydro-5,6-dehydrokavain (DDK), a powerful anti-inflammatory and antioxidative agent, which may be a promising therapy for patients with NAFLD patients; however, it requires further research [98,99].

Cocoa has gained interested based on its antioxidative potential and has been found to decrease NADPH oxidase levels in NASH patients and reduce oxidative stress [100,101]. Similarly, cinnamon has been studied for its antioxidative effects as well and a blinded trial in NAFLD patients by Askari et al. reported that 1500 mg of cinnamon supplementation for 12 weeks reduced AST, ALT, and insulin resistance compared to the placebo group [102].

## 5. Traditional Chinese Herbs

### 5.1. Ginkgo Biloba

*Ginkgo biloba* is one of the most common trees in China and its leaves have been studied for their therapeutic properties [103,104,105,106,107,108]. Through the upregulation of CPT-1A, ginkgo biloba can regulate fatty acid metabolism in the liver and reduce fat accumulation as well as decrease ROS production [103,104]. Furthermore, the ability of *Gingko biloba* to increase antioxidative enzymes SOD, GPx, and catalase allow it to reduce the oxidative stress in the liver as well [106]. Multiple experimental studies have been completed in NAFLD- and NASH-induced mice with *Gingko biloba* extract supplementation and noted improved AST, ALT, hepatic inflammation, and hepatic steatosis [103,104,105,106,107,108].

### 5.2. Ginseng

Ginseng, or *Panax ginseng*, has been often used in traditional Chinese medicine to treat multiple metabolic conditions, including hepatosteatosis [52,109,110,111,112,113]. The active agents in ginseng are ginsenosides and saponins [109,111,112,113]. Ginseng is able to reduce lipid accumulation through the induction of the SIRT1/AMPK pathway and activate auto-phagocytic pathways [111,112]. Moreover, ginseng is able to reduce hepatic fibrosis factors such as collagen-1 and α-SMA and prevent HSC activation [113]. Ginseng is able to also reduce inflammation and ER stress through GRP78 [113]. Multiple mouse studies observed improved steatosis, liver enzymes, hepatic inflammation, and fibrosis in NAFLD/NASH-induced mice supplemented with ginseng extract [109,110,111,112,113]. Current data on ginseng supplementation seems promising for future trials and use with NAFLD patients.

### 5.3. Lotus, Goji Berry, Astralagus, and Ciruwujia

These four herbs have been used in traditional Chinese medicine and have recently started being used in experimental studies which have indicated their potential for therapeutic use in NAFLD through improved liver function, steatosis, and inflammation in mice [114,115,116,117,118,119,120,121,122,123,124,125,126]. However, there are no clinical trials supporting their use. Lotus can be used to exert anti-inflammatory effects on the liver through its regulation of IL-6, NF-κB, TNF-α, and TGF-β1 [115,118,119,123]. Also, through its regulation of adiponectin, lotus can promote hepatic lipid homeostasis [115,118,119,123]. Goji berry, from the plant *Lycium barbarum*, exerts its hepatoprotective effects through regulation of lipid homeostasis (SIRT1/AMPK and SREBP-1c), and inflammation (NF-κB) [116,117,120,125]. Astragalus and ciruwujia have yet to be fully explored as therapeutic agents, but regression of fatty liver in NAFLD-induced mouse studies warrant more in-depth understanding [114,121,122,124,126]. Astragalus has hypothesized to function through CPT-1, FXR, PPARα, and SREBP-1c to give the its anti-inflammatory and lipid homeostatic effects to improve liver function and steatosis in mice [122,124,126]. Ciruwujia has been postulated to improve liver function through IL-6, CPT-1α, and TLR4 to confer anti-inflammatory effects [114,121].

## 6. Other Herbs

### 6.1. Cannibinoids

Cannabinoids have gained interest on their effects due to controversy of their usage across the United States and mixed understanding on their possible therapeutic effects on NAFLD [127,128,129,130,131,132,133,134]. A cross-sectional study by Adejumo et al. noted a statistically significant lower prevalence of NAFLD in cannabis users and another larger study by Kim et al. concluded an inverse relationship between cannabis use and suspected and ultrasonographically-diagnosed NAFLD [132,133]. Further research found that cannabinoids act through two receptors—cannabinoid receptors 1 and 2 (CB1 and CB2) [127,128,129,134]. In general, CB1 is pro-fibrogenic, while CB2 is anti-fibrogenic [127,128,129,134]. Tetrahtydrocannabinol (THC) acts via both receptors in a dose-dependent relationship, in which at low-levels it is a CB1 antagonist and has therapeutic effects, but at higher levels becomes a CB1 agonist and becomes a risk factor [127,128,129,134]. Further research on the exocannabinoid and endocannabinoid system is warranted to fully understand their possible therapeutic use.

### 6.2. Licorice, Red Clover, and Chamomile

Licorice, red clover, and chamomile are herbs currently being explored for their possible therapeutic use and are possible routes for future research. Licorice has been proposed to promote lipid homeostasis through SREBP-1c, FAS, ACC1, PPARα, and CPT-1α [135,136]. A double-blinded clinical trial by Hajiaghamohammadi et al. randomized 66 patients to receive either placebo or 2 g aqueous licorice root extract for 2 months and observed improved AST and ALT in the treated group [137]. Red clover and chamomile are hypothesized to have hepatoprotective effects due to their possible role in PPARα regulation, but currently not enough is known about how they function in terms of NAFLD and more studies are necessary [138,139] Table 1 summarizes the various herbs studied in NAFLD therapy.

## 7. Non-Herb Supplements

### 7.1. Fish Oils

Fish oils, mainly eicosapentaenoic acid (EPA) and docosahexanoic acid (DHA), represent one of the most highly marketed and widely used supplement types for many metabolic conditions [140,141,142,143,144,145,146,147,148,149,150,151,152,153]. They can improve lipid homeostasis and inflammation through the PPARα, AMPK, and adiponectin [140,141,142,143,144,145,146,147,148,149,150,151,152,153]. A 41-patient NASH study by Argo et al. randomized patients to receive 3000 mg/day of fish oil or placebo for 1 year and observed improved ALT in the treated patients [140]. Another study by Boyraz et al. in 52 obese NAFLD patients treated with 1000 mg fish oil reported improved AST, ALT, and steatosis [141] Nogueira et al. noted that fish oil supplementation improved steatosis and inflammation in patients [147]. Meta-analysis by Yu et al. calculated that fish oil supplementation improves ALT [152]. Another meta-analysis by Chen et al. noted that fish oil supplementation in NAFLD children improves steatosis, ALT, and AST [153].

### 7.2. Coenzyme Q-10

Like fish oil, coenzyme Q-10 (CoQ10) is commonly seen in stores and used widely. Many mouse studies have noted the hepatoprotective effects of CoQ10 [154,155,156]. A study by Farhangi et al. in NASH-induced mice noted improved liver biochemical enzymes after supplementation [155]. CoQ10 is able to modulate TNF-α and adiponectin levels to improve inflammation and lipid homeostasis in the liver [155,157]. A blinded RCT by Farsi et al. treated NAFLD patients with 100 mg CoQ10 and noted reduced AST, ALT, and hepatic inflammation compared to placebo [157].

## 8. Safety Concerns for Herb Supplementation

Despite the apparent therapeutic effects of the herbs discussed in this article, it is important to keep in mind that there are no guidelines or recommendations for herb usage in NAFLD management [2]. The lack of regulation in this field of treatment agents suggests caution with use of herbs in the context of NAFLD. Also, the lack of research indicates the possibility of drug interactions or adverse effects [158,159,160,161]. Herb–drug interactions have already been noted in warfarin, aspirin, alkylating agents, and cyclosporine, leading to toxicity in patients [158,161]. Furthermore, as natural herbs are converted to supplements, the final product may no longer be purely herbal and can contain trace metals that could become toxic if taken consistently at high levels [162]. Alhusban et al. discovered that some commonly used pharmaceutical herbal products contained toxic metals, such as Pb, Al, Ni, Cd, and As [162]. Of these, some products had recommended intakes that led to accumulation of the toxic metals greater than the tolerable daily intake set by the Food and Drug Administration (FDA) [162].

Furthermore, some studies have indicated that some herbs may have hepatotoxic characteristics [163,164,165,166]. Multiple studies have noted herb-induced liver injury (HILI), in particular with Chinese herbal medicine [163,164,165,166]. However, there have been obstacles in accurately assessing if liver injury is truly herb-induced versus drug-induced; more accurate diagnostic tools are currently being developed to make this distinction [163,164,165,166]. Nonetheless, the possible presence of hepatotoxins in some herbs and their unregulated, non-standardized use can lead to worsening of underlying liver disease, NASH, or other chronic hepatic diseases. Also, none of the studies reviewed noted obesity as a contraindication of herb usage. Extensive research is necessary to test the safety of herbs as a therapeutic agents before their use can be recommended in patients with NAFLD or other ailments.

## 9. Conclusions

All in all, there are many promising herbs and supplements that can be used as an alternative treatment for NAFLD. Some of them are basic nutrients that people use daily as spices and seasonings in their diet. However, to say that they should be part of a treatment for NAFLD and/or NASH is to overstep, as there is there is no clinical data that support this notion. Milk thistle, turmeric, resveratrol, coffee, and green tea have been extensively researched in the NAFLD population and have not been shown to have toxic effects. We can only recommend that they be used with caution and that further research is necessary to confirm the therapeutic role of herbs in patient with NAFLD.

## Figures and Tables

**Table 1:** The extent of research for each herb in the setting of nonalcoholic fatty liver disease (NAFLD) therapy.

Herb	Level of Research with NAFLD Context	Mechanism of Action
Milk thistle [6,7,8,9,10,11,12,13,14,15,16,17,18,19,20,21,22,23,24,25]	Experimental and clinical studies (RCTs)	Antioxidative Anti-apoptotic Anti-inflammatory Anti-adipogenic
Resveratrol [26,27,28,29,30,31,32,33,34,35,36]	Experimental and clinical studies (RCTs)	Antioxidative Anti-apoptotic Anti-adipogenic
Coffee [37,38,39,40,41,42,43,44,45,46,47,48,49,50]	Experimental and clinical studies (CSs, CCs)	Antioxidative Anti-apoptotic Anti-inflammatory Anti-fibrotic
Green tea [51,52,53,54,55,56,57,58,59,60,61,62,63]	Experimental and clinical studies (RCTs)	Antioxidative Anti-inflammatory
Turmeric [64,65,66,67,68,69,70,71,72,73,74,75,76,77,78]	Experimental and clinical studies (RCTs)	Antioxidative Anti-apoptotic Anti-inflammatory Anti-adipogenic
Garlic [79,80,81,82,83,84,85,86]	Experimental and clinical studies (RCTs)	Antioxidative Anti-apoptotic Anti-inflammatory
Ursolic acid (rosemary, peppermint, basil, lavender, and oregano) [87,89,90]	Experimental studies	Anti-inflammatory Anti-adipogenic
Carnosic acid (rosemary and sage) [88,91,92,93,94]	Experimental studies	Antioxidative Anti-apoptotic Anti-inflammatory Anti-adipogenic
Ginger [95,96,97]	Experimental and clinical studies (RCTs)	Antioxidative Anti-apoptotic Anti-inflammatory Anti-adipogenic
Cocoa [100,101]	Experimental studies	Antioxidative
Cinnamon [102]	Clinical studies (RCTs)	Antioxidative
*Ginkgo biloba* [103,104,105,106,107,108]	Experimental studies	Antioxidative Anti-apoptotic Anti-inflammatory Anti-adipogenic
Ginseng [52,109,110,111,112,113]	Experimental and clinical studies (RCTs)	Anti-inflammatory Anti-adipogenic Anti-fibrotic
Lotus [115,118,119,123]	Experimental studies	Anti-inflammatory
Goji Berry [116,117,120,125]	Experimental studies	Anti-inflammatory Anti-adipogenic
Astragalus [122,124,126]	Experimental studies	Anti-inflammatory Anti-adipogenic
Ciruwuijia [114,121]	Experimental studies	Anti-inflammatory
Cannabinoids [127,128,129,130,131,132,133,134]	Experimental and clinical studies (CSs)	Anti-fibrotic
Licorice [135,136,137]	Experimental and clinical studies (RCTs)	Anti-inflammatory
Red clover [138]	Experimental studies	Anti-inflammatory
Chamomile [139]	Experimental studies	Anti-inflammatory

Abbreviations: randomized control trial (RCT), cross-sectional study (CS), case–control study (CC).

## Abbreviations

NAFLD	nonalcoholic fatty liver disease
NASH	nonalcoholic steatohepatitis
PARP	poly-(ADP-ribose)-polymerase
PPARγ	peroxisome proliferator activated receptor γ
ACC	acetyl-CoA carboxylase
FAS	fatty acid synthase
FXR	farnesyl X receptor
NAS	NAFLD activity score
ALT	alanine amino transferase
AST	aspartate amino transferase
ER	endoplasmic reticulum
CHOP	C/EBP homologous protein
CPT-1	carnitine palmitoyltransferase
UCP-2	uncoupling protein
ROS	reactive oxygen species
GRP78	glucose-related protein 78
PRDX-1	periredoxin-1
EGCG	epigallocatechin-3-gallate
COX2	cyclooxygenase-2
TLR4	toll-like receptor 4
CYP2E1	cytochrome P450 2E1
Nrf2	nuclear factor E2 related factor
CREB	cAMP response element-binding
SAC	S-allyl cysteine
SAMC	S-allymercaptocysteine
DADS	diallyl disulfide
GPx	glutathione peroxidase
HSCs	hepatic stellate cells
SOD	superoxide dismutase
DDK	dihydro-5,6-dehydrokavain
CB1	cannabinoid receptor 1
CB2	cannabinoid receptors 2
EPA	eicosapentaenoic acid
DHA	docosahexanoic acid
CoQ10	coenzyme Q-10
FDA	Food and Drug Administration
HILI	herb-induced liver injury
